# A Comparison of Diagnostic Methods for Feline Leukemia Virus and Feline Immunodeficiency Virus: Immunochromatographic Assay and RNases Hybridization-Assisted Amplification Test Kit Compared to Reverse Transcription Quantitative Polymerase Chain Reaction

**DOI:** 10.3390/ani15101484

**Published:** 2025-05-20

**Authors:** Thanikran Suwannachote, Wisut Prasitsuwan, Thirawat Sumalai, Sakchai Ruenphet

**Affiliations:** 1 Clinic for Small Domestic Animals and Radiology, Mahanakorn University of Technology, Bangkok 10530, Thailand; dv15006a@gmail.com; 2 Animal Biotechnology, Mahanakorn University of Technology, Bangkok 10530, Thailand; vizutrto@gmail.com (W.P.); vet47ku@gmail.com (T.S.); 3 Immunology and Virology Department, Mahanakorn University of Technology, Bangkok 10530, Thailand

**Keywords:** feline leukemia virus, feline immunodeficiency virus, immunochromatographic assay, reverse transcription quantitative polymerase chain reaction, RNase hybridization-assisted amplification

## Abstract

Feline leukemia virus (FeLV) and Feline immunodeficiency virus (FIV) are globally prevalent retroviral pathogens that pose significant health risks to domestic cats. This study aimed to compare the diagnostic performance of two point-of-care—the immunochromatographic assay (ICA) and the RNase hybridization-assisted amplification (RHAM) test kit—against reverse transcription quantitative polymerase chain reaction (RT-qPCR), the current gold standard for FeLV and FIV detection. For FeLV detection, ICA demonstrated a sensitivity of 86.89%, specificity of 96.55%, accuracy of 90.00%, and precision of 98.15%, while for FIV detection, the assay showed a sensitivity of 75.86%, specificity of 88.52%, accuracy of 84.44%, and precision of 75.86%. In contrast, the RHAM test exhibited superior performance, with FeLV detection sensitivity of 93.44%, specificity of 98.28%, accuracy of 94.44%, and precision of 98.28%. For FIV detection, RHAM demonstrated a sensitivity of 75.86%, specificity of 100%, accuracy of 92.22%, and precision of 100%. Additionally, the RHAM assay significantly reduced detection time compared to RT-qPCR, enabling expedited clinical decision-making, alleviating laboratory workload, and lowering diagnostic costs. These benefits are particularly relevant in veterinary settings with limited access to PCR-based diagnostics, where the RHAM assay represents a rapid, reliable, and resource-efficient alternative for FeLV and FIV detection.

## Simple Summary

RNase hybridization-assisted amplification (RHAM) is a novel nucleic acid detection technology that integrates loop-mediated isothermal amplification (LAMP)-mediated exponential amplification with an RNase HII reporter system, enabling signal visualization within a single reaction. In this study, a field trial demonstrated that the RHAM test kit effectively detects feline leukemia virus (FeLV) and feline immunodeficiency virus (FIV), exhibiting high sensitivity, specificity, accuracy, and precision when compared with reverse transcription quantitative polymerase chain reaction (RT-qPCR), the current gold standard diagnostic method. The trials confirmed the RHAM kit’s reliability in detecting FeLV and FIV nucleic acid in feline samples. Furthermore, the RHAM FeLV/FIV test kit significantly reduced detection time compared to RT-qPCR, thereby decreasing laboratory workload and expediting result reporting. Consequently, the RHAM test kit represents a rapid, convenient, and reliable tool for FeLV and FIV detection, offering diagnostic performance comparable to RT-qPCR. These advantages, including shorter turnaround times and reduced resource requirements, make RHAM particularly advantageous for use in veterinary settings with limited access to advanced PCR-based diagnostics.

## 1. Introduction

Feline leukemia virus (FeLV) and feline immunodeficiency virus (FIV) are retroviruses that infect domestic cats worldwide. Both FeLV and FIV are transmitted horizontally through saliva, body fluids, close contact, mutual grooming, sharing of equipment and food, as well as through scratching and biting [1,2]. Maternal transmission from mother to kitten has also been documented [3,4,5]. The pathogenicity of FeLV and FIV varies due to factors such as viral subtype, host immune response, and the presence of co-infections, all of which influence disease progression and severity [6]. In practice, FeLV screening is commonly performed using test kits that detect p27, a critical core protein in the virus. Enzyme-linked immunosorbent assay (ELISA) and immunochromatographic assay (ICA) are routinely used for the serodiagnosis of FeLV and FIV [7,8]. However, polymerase chain reaction (PCR) confirmation is essential for validating positive results, as initial screening tests such as ELISA and ICA may occasionally produce false positives or false negatives or yield inconclusive results due to factors such as sample handling and variations in test sensitivity. PCR, known for its high specificity, serves as a confirmatory method that accurately distinguishes true infections from false diagnoses, ensuring the reliability and precision of results for clinical decision-making [9,10,11].

FeLV is a gammaretrovirus within the family Retroviridae, characterized by its ability to integrate into the host genome. Certain retroviral sequences, collectively referred to as endogenous FeLV (enFeLV), are stably integrated into the feline germline and inherited vertically. In contrast, exogenous FeLV (exFeLV) arises in infected cats through the activation and recombination of these endogenous elements with exogenous viral RNA during infection events [12]. ExFeLV is further classified into distinct subgroups—FeLV-A, FeLV-B, and FeLV-C—based on genetic divergence and functional receptor usage [13,14]. Among these, FeLV-A is the predominant subgroup detected in naturally infected cats, while FeLV-B and FeLV-C generally arise secondarily via recombination between exogenous FeLV-A and endogenous sequences following primary infection [15,16]. Detection of free viral p27 capsid antigen in peripheral blood remains the most common method for identifying FeLV antigenemia in viremic cats [8,17]. Although point-of-care tests targeting p27 offer high analytical sensitivity, they may exhibit reduced specificity, potentially leading to false-negative or ambiguous results. Therefore, molecular confirmation using PCR-based assays targeting viral RNA or integrated proviral DNA is recommended to enhance diagnostic accuracy [18,19]. Notably, quantitative PCR is capable of detecting all FeLV subgroups by amplifying conserved regions shared across the proviral genomes of FeLV-A, FeLV-B, and FeLV-C.

FIV is a retroviral pathogen classified within the genus Lentivirus of the Retroviridae family and is characterized by its ability to integrate into the host genome [20,21]. The FIV genome comprises three principal–structural genes—*gag*, *pol*, and *env*—as well as multiple accessory genes that contribute to viral replication and pathogenesis. Phylogenetic analyses based on the *env* and *gag* gene regions have identified at least seven distinct FIV subtypes [22,23]. Infected cats may remain asymptomatic for a prolonged period; however, FIV infection can ultimately lead to acquired immunodeficiency, increasing susceptibility to opportunistic infections and other clinical complications. Diagnosis typically relies on the detection of virus-specific neutralizing antibodies or identification of the viral genome (RNA or proviral DNA) [24]. It is important to note that serological tests may produce false-negative results during the early stages of infection, particularly within the first 60 days post-exposure [25]. To improve diagnostic sensitivity and specificity, PCR-based assays are commonly employed and are designed to target highly conserved regions of the viral genome, such as the *gag* gene [26].

A novel diagnostic method, RNase hybridization-assisted amplification (RHAM), has recently been developed, integrating loop-mediated isothermal amplification (LAMP) with RNase HII to enable the rapid detection of specific viral targets for FeLV and FIV. The objectives of this study were: (i) to assess the prevalence of FeLV and FIV infections in sick cats treated at three veterinary hospitals in Bangkok, Thailand; (ii) to compare the diagnostic accuracy of immunochromatographic assay (ICA) for detecting FeLV antigens and FIV antibodies with that of reverse transcription quantitative PCR (RT-qPCR); (iii) to evaluate the performance of the RHAM method for detecting FeLV and FIV in comparison to RT-qPCR.

## 2. Materials and Methods

### 2.1. Ethical Approval and Informed Consent

The study was approved by the Animal Research Ethics Committee of the Faculty of Veterinary Medicine at Mahanakorn University of Technology, Thailand (approval number ACUC-MUT-2024/013). In addition, written informed consent was obtained from the owners of the cats for their pets’ participation in the study.

### 2.2. Study Period and Location

Between December 2024 and February 2025, blood samples were collected for analysis from three veterinary facilities in Bangkok, Thailand: The Small Animal Teaching Hospital at the Faculty of Veterinary Medicine, Mahanakorn University of Technology; Kling Kaew Animal Hospital; and the Vet Home Polyclinic.

### 2.3. Sample Collection

Ninety whole blood samples were collected by a licensed veterinarian from cats of varying ages and breeds, presenting with clinical signs suggestive of infection. Blood samples were collected in EDTA tubes and immediately tested using an ICA. The remaining samples were stored at −80 °C for subsequent analysis using the RHAM test and RT-qPCR, with duplicate testing conducted for accuracy. All cats exhibited clinical signs consistent with FeLV or FIV infections, including fever, anemia, weight loss, diarrhea, and pale mucous membranes, as determined during physical examination. To minimize the potential influence of maternal antibodies, kittens under six months of age that tested positive for FIV were excluded from the study.

### 2.4. Immunochromatographic Assay (ICA)

A commercial lateral flow immunochromatographic test with colloidal gold (VetDiag^®^, Pacific Biotech, Petchaboon, Thailand) was utilized to detect infections with FeLV and FIV. This test was performed and interpreted according to the manufacturer’s instructions.

### 2.5. RNase Hybridization-Assisted Amplification (RHAM) Test Kit

**Figure 1:**
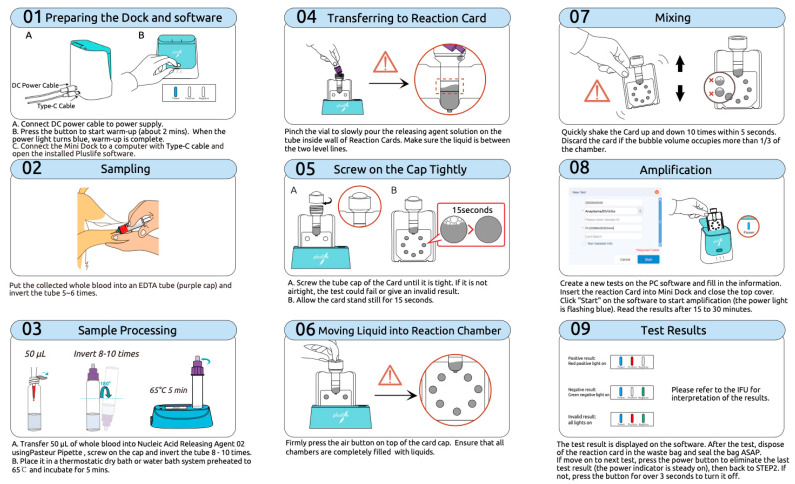
Schematic representation of the operational workflow for the RNase hybridization-assisted amplification (RHAM) test kit used for nucleic acid detection of feline leukemia virus (FeLV) and feline immunodeficiency virus (FIV). This illustration outlines the standardized step-by-step procedure for detecting FeLV and FIV DNA using a closed nucleic acid test card system. *Step 1*: Connect the Mini Dock device to a power source and USB interface, then launch the operating software. *Step 2*: Collect whole blood into an EDTA tube and gently invert 5–6 times. *Step 3*: Mix 50 µL of whole blood with Nucleic Acid Releasing Agent, invert 8–10 times, and incubate at 65 °C for 5 min. *Step 4*: Transfer the resulting lysate into the designated well of the reaction card. *Step 5*: Seal the card securely with the cap and allow it to stand undisturbed for 15 s. *Step 6*: Press the air chamber button to ensure complete filling of the reaction chambers. *Step 7*: Mix the contents thoroughly by manual shaking. *Step 8*: Insert the card into the Mini Dock and initiate the amplification process via the software interface. *Step 9*: Interpret the results as displayed on the software platform.

The RHAM test kit for the detection of FeLV and FIV nucleic acid comprises three primary components: the Pluslife Integrated Nucleic Acid Testing Device, a nucleic acid releaser tube, and the Pluslife FeLV/FIV Nucleic Acid Test card, all patented by Guangzhou Pluslife Biotech, China. The testing process begins by preheating the device and establishing a connection with the Pluslife Pet Application via a mobile phone or computer. Following preheating, 50 μL of whole blood is collected using a disposable Pasteur pipette and transferred into the nucleic acid releaser tube. The tube is then incubated at 65 °C for 5 min to facilitate nucleic acid release. Subsequently, the lysate is transferred to the designated inlet on the FeLV/FIV test card. The card is left to stand upright for 15 s before activating the air button on the cartridge cap to ensure proper reagent distribution. The card is briefly shaken and then inserted into the testing device. Amplification is initiated by selecting the “Start” function on the software or mobile application, with results available for interpretation after approximately 30 min. A detailed schematic of the entire workflow is presented in Figure 1.

### 2.6. Reverse Transcription Quantitative Polymerase Chain Reaction (RT-qPCR)

Genomic DNA and RNA extractions for the detection of FeLV and FIV were performed using the TAN Bead^®^ Nucleic Acid Extraction kit (Taiwan Advanced Nanotech, Taoyuan, Taiwan) in accordance with the manufacturer’s protocols. The extraction procedure was conducted utilizing the Automated Nucleic Acid Extractor (Smart LabAssist SLA-E13200, Taiwan Advanced Nanotech, Taiwan). Following nucleic acid extraction, detection of FeLV and FIV genomes was carried out using the Primerdesign genesig kit (Primerdesign^TM^ Ltd., Hampshire, UK) in combination with the C1000 Touch Thermal Cycler (Bio-Rad Laboratories, Berkeley, CA, USA). The one-step RT-qPCR protocol employed included an initial reverse transcription step at 55 °C for 10 min, followed by enzyme activation at 95 °C for 2 min, and 50 amplification cycles comprising denaturation at 95 °C for 10 s and annealing/extension at 60 °C for 60 s. The results were interpreted based on cycle threshold (Ct) values, with samples exhibiting Ct values below 50 classified as positive, while those exceeding 50 were deemed negative.

### 2.7. Analysis

The prevalence of FeLV and FIV infections in the study population was calculated and expressed as percentages to provide an overview of infection rates. The diagnostic performance, including sensitivity, specificity, accuracy, and precision of the evaluated methods, was determined by comparing the results obtained from ICA or RHAM with those from RT-qPCR, which served as the reference standard. Results concordant with RT-qPCR were classified as true positives or true negatives, while discordant results were considered false negatives or false positives. The respective numbers of true positives, false positives, true negatives, and false negatives for ICA or RHAM, in comparison to RT-qPCR, were documented for subsequent analysis. Sensitivity, specificity, accuracy, and precision for each diagnostic modality were calculated using standard formulas derived from the evaluation data:

$$Sensitivity=\frac{No.of\;true\;positive}{No.of\;true\;positive+No.of\;false\;negative}\times 100,$$

$$Specificity=\frac{No.of\;true\;negative}{No.of\;true\;negative+No.of\;false\;positive}\times 100,$$

$$Accuracy=\frac{\left(No.of\;true\;positive\right)+(No.of\;true\;negative)}{\left(No.of\;true\;positive\right)+\left(No.of\;true\;negative\right)+\left(No.of\;false\;positive\right)+(No.of\;false\;negative)}\times 100,$$

$$Precision=\frac{No.of\;true\;positive}{No.of\;true\;positive+No.of\;false\;positive}\times 100$$

For all diagnostic methods evaluated, 95% confidence intervals (CIs) for sensitivity, specificity, accuracy, and precision were calculated using the Wilson score interval method for binomial proportions. This approach is preferred due to its accuracy and reliability in estimating CIs, particularly when dealing with proportions derived from binary diagnostic outcomes, such as positive or negative test results.

## 3. Results

The prevalence of FeLV and FIV among 90 whole blood samples collected from clinically ill cats in Bangkok, Thailand, during the winter season of 2024–2025, was determined to be 60.00% (54/90) for FeLV antigen positivity and 32.22% (29/90) for FIV antibodies. Using the RHAM assay, FeLV was detected in 64.44% (58/90) of samples and FIV in 24.44% (22/90). In comparison, RT-qPCR identified FeLV in 67.78% (61/90), FIV in 32.22% (29/90), and co-infection in 21.11% (19/90) of the samples. The raw data comparing ICA with RT-qPCR for FeLV and FIV detection are presented in Table 1 and Table 2, respectively. Similarly, Table 3 and Table 4 display the comparative data between RHAM and RT-qPCR for FeLV and FIV detection. A consolidated summary of these findings is provided in Table 5 for clarify.

The diagnostic performance of the ICA and RHAM tests was evaluated by calculating sensitivity, specificity, accuracy, and precision, with the corresponding 95% confidence intervals (CIs) presented in Table 6. For the ICA test, the sensitivity for FeLV detection was 86.89% (95% CI: 82.24–90.91%), specificity was 96.55% (95% CI: 94.34–98.04%), accuracy was 90.00% (95% CI: 85.96–92.82%), and precision was 98.15% (95% CI: 95.67–99.19%). For FIV detection, the sensitivity was 75.86% (95% CI: 66.25–84.15%), specificity was 88.52% (95% CI: 82.32–93.72%), accuracy was 84.44% (95% CI: 79.24–89.44%), and precision was 75.86% (95% CI: 66.25–84.15%) (Table 6).

For the RHAM assay, the sensitivity for FeLV detection was 93.44% (95% CI: 90.12–96.11%), specificity was 98.28% (95% CI: 96.22–99.35%), accuracy was 94.44% (95% CI: 91.22–96.32%), and precision was 98.28% (95% CI: 96.22–99.35%). Regarding FIV detection, the sensitivity was 75.86% (95% CI: 66.25–84.15%), specificity was 100% (95% CI: 100–100%), accuracy was 92.22% (95% CI: 88.61–94.72%), and precision was 100% (95% CI: 100–100%) (Table 6).

## 4. Discussion

Infections with FeLV and FIV are globally prevalent and pose significant health risks to domestic cats. Given the potential clinical implications of these infections, the objective of the present study was to determine the prevalence of FeLV and FIV among clinically ill cats presented for treatment at three small animal hospitals in Bangkok, Thailand. In addition to assessing prevalence, the study aimed to evaluate the diagnostic performance of two alternative methods in comparison to the gold standard, RT-qPCR, for the detection of FeLV and FIV infections. Using RT-qPCR as the reference standard, the prevalence rates of FeLV and FIV were identified as 67.78% and 32.22%, respectively, with 21.11% of cats testing positive for co-infections with both viruses. These findings indicate a notably high prevalence of FeLV and FIV among the clinically ill cats included in this investigation. In contrast, Rungsuriyawiboon et al. [27] reported considerably lower prevalence rates of 12.50% for FeLV, 8.30% for FIV, and 2.70% for co-infections, based on a routine health monitoring survey conducted at Kasetsart University, Bangkok, Thailand. Similarly, Sprißler et al. [8] reported FeLV and FIV prevalence rates of 4.20% and 5.80%, respectively, with a co-infection rate of 0.40%, in a study conducted at seven sites in Bangkok and six locations across northern, northeastern, and central Thailand. Their study population comprised cats enrolled in preventive health care and neutering programs. The higher prevalence observed in the present study is likely attributable to the fact that the sampled population consisted of clinically ill cats, whereas the studies conducted by Rungsuriyawiboon et al. [27] and Sprißler et al. [8] focused on routine health monitoring and preventive care cohorts.

Infection with FIV results in the integration of provirus DNA into the host genome, establishing a lifelong infection. The persistence of the virus is a critical factor in disease progression, which is classically divided into three distinct phases. The initial phase, referred to as primary infection, is characterized by viremia, during which affected cats may exhibit transient clinical signs such as malaise or peripheral lymphadenopathy. This is followed by the asymptomatic phase, the longest stage of infection, wherein viral replication is markedly reduced, and cats generally remain clinically healthy. The terminal phase is marked by a resurgence of viral replication, resulting in overt clinical disease, which is partly associated with progressive CD4+ lymphocytopenia [28]. FIV induces a robust and persistent antibody response, detectable from the primary phase and persisting throughout the course of infection. As such, antibody detection remains the cornerstone of FIV diagnosis in clinical settings [19]. However, in the present study, 7 samples tested positive for FIV antibodies while nucleic acid detection using both RHAM and RT-qPCR was negative (Table 2). The absence of detectable viral nucleic acid in these samples may reflect false-positive antibody results from the ICA or indicate that the cats were in the asymptomatic phase, during which viral replication is minimal and clinical signs are absent. Additionally, prior vaccination with an inactivated FIV vaccine is a known cause of false-positive antibody results, as vaccinated cats may exhibit persistent antibody titers despite being uninfected. Before the introduction of the FIV vaccine, diagnosis was straightforward using point-of-care antibody tests, which were simple, cost-effective, and exhibited high diagnostic accuracy, comparable to Western blot analysis. This approach was particularly effective in regions such as Australia, New Zealand, and Japan, where the vaccine has been widely adopted [29,30]. However, following the introduction of the vaccine, studies have demonstrated that vaccinated cats consistently tested antibody-positive irrespective of infection status, thereby raising concerns regarding the reliability of antibody-based diagnostics in vaccinated populations [31,32]. To address this limitation, the present study employed both antibody and nucleic acid detection methods to differentiate vaccinated from naturally infected cats.

Progressively infected FeLV-positive cats differ fundamentally from FIV-infected cats in that they exhibit persistently high circulating viral loads accompanied by an inconsistent antibody response. Due to the unreliability of this antibody response, FeLV infection is primarily diagnosed through the detection of viral antigen rather than antibodies [19]. Specifically, all currently available point-of-care tests for FeLV target the viral capsid protein p27, a critical biomarker of active infection [33]. Conversely, FeLV antibody testing is generally limited to research settings, where it is employed to assess historical exposure rather than current infection status. The evaluation of FeLV point-of-care test kit performance is further complicated by the absence of a universally accepted gold standard for FeLV diagnosis [29,30,34]. In light of these diagnostic challenges, the present study aimed to assess the efficacy of a point-of-care nucleic acid amplification test. Accordingly, the diagnostic performance of the RHAM point-of-care nucleic acid test kit for FeLV detection was evaluated and compared with RT-qPCR, which served as the reference standard. The results demonstrated that the RHAM assay exhibited high sensitivity, specificity, accuracy, and precision, surpassing the performance of ICA-based antigen detection, particularly with respect to sensitivity and specificity (Table 6).

The RHAM FeLV/FIV test kit utilizes isothermal amplification combined with enzyme digestion probe technology to detect and identify specific target sequences of both viruses. To meet the demand for a versatile and rapid diagnostic platform, the RHAM assay incorporates an innovative isothermal amplification approach that integrates loop-mediated isothermal amplification (LAMP) with an RNase HII-mediated reporter system, enabling signal visualization within a single reaction chamber. Initially, a conventional LAMP primer set facilitates the exponential amplification of the target nucleic acid sequences using Bst DNA polymerase. Following amplification, a dual-labeled fluorescent probe—bearing a fluorophore at the 5′-end and a quencher at the 3′-end—hybridizes to the amplified product. Within this probe-target duplex, RNase HII specifically recognizes and cleaves the ribonucleotide site, releasing the fluorophore from the quencher and generating a detectable fluorescence signal [35,36,37]. This integrated platform enables fluorescence detection within 30 min, offering rapid and efficient detection of FeLV and FIV. Moreover, the assay incorporates an internal control to monitor the entire diagnostic workflow, including sample collection, processing, and amplification steps, thereby minimizing the risk of false-negative and false-positive results and enhancing overall test reliability. In the present study, the RHAM test kit demonstrated high sensitivity, specificity, accuracy, and precision when benchmarked against RT-qPCR. Furthermore, 95% CIs were calculated for these diagnostic parameters for both the ICA and RHAM assays, providing a more comprehensive evaluation of their diagnostic performance. The narrow CIs observed for both methods indicate minimal variability and high test reliability, with RHAM consistently outperforming ICA, particularly in sensitivity and specificity. The overlapping Cls for certain parameters, such as specificity, further corroborate the robustness of both diagnostic approaches, underscoring their potential utility in routine clinical settings. Importantly, the inclusion of CIs enhances the statistical validity of the findings, supporting the conclusion that the RHAM assay represents a reliable and efficient alternative to RT-qPCR, particularly suited for use in resource-limited environments.

The accurate identification of cats infected with FeLV or FIV is critical for the control and management of these potentially fatal pathogens. Consequently, point-of-care rapid test kits are widely employed for infection screening, as test outcomes can substantially influence clinical decision-making. In this study, the diagnostic performance of a lateral flow immunochromatographic assay (ICA) and a novel nucleic acid-based test kit (RHAM) was evaluated and compared with RT-qPCR for the detection of FeLV and FIV in cats. The RHAM assay demonstrated superior sensitivity, specificity, accuracy, and precision for FeLV detection compared to ICA, as validated by RT-qPCR reference testing. For FIV detection, RHAM outperformed ICA across most parameters, with the exception of sensitivity, where the difference was not statistically significant, as reflected in the study’s findings (Table 6). Despite its overall strong performance, the RHAM assay yielded four false-negative results for FeLV detection in samples with RT-qPCR cycle threshold (Ct) values exceeding 32.08 (Table 3), and seven false-negative results for FIV detection in samples with Ct values above 30.43 (Table 4). These observations suggest a reduced sensitivity of the RHAM assay in samples with very low pathogen loads. Additionally, the RHAM test utilized only 50 μL of sample—approximately one-sixth of the volume used in RT-qPCR—which may have contributed to the lower sensitivity observed in low-titer samples. A single false-positive result was recorded for FeLV detection, likely attributable to cross-contamination during sample handling or laboratory procedures, underscoring the need for rigorous procedural controls. Overall, these results demonstrate that while the RHAM assay offers a rapid, convenient, and accurate approach for FeLV and FIV detection, it cannot entirely replace RT-qPCR, particularly in cases of low viral load. Nonetheless, the RHAM test kit, introduced in late 2024, represents the first reported application of this technology in feline diagnostics, highlighting its potential as a valuable tool for routine clinical use.

## 5. Conclusions

The RHAM assay for the detection of FeLV and FIV offers several advantages over conventional diagnostic methods, including the provision of rapid results within 30 min, thereby facilitating timely clinical decision-making, particularly in high-throughput settings or resource-limited environments. The assay demonstrates high sensitivity, specificity, accuracy, and precision, comparable to those achieved with RT-qPCR, while obviating the need for sophisticated laboratory infrastructure, thus representing a cost-effective and portable diagnostic alternative. Nonetheless, the RHAM test has certain limitations, including the occurrence of occasional false-negative results, particularly in samples with low viral loads, and a reduction in sensitivity attributable to the smaller sample volume utilized. Despite these limitations, the RHAM assay remains a promising and practical alternative for field diagnostics and point-of-care testing applications.

## Figures and Tables

**Table 1:** Comparative raw data of immunochromatographic assay (ICA) and reverse transcription quantitative polymerase chain reaction (RT-qPCR) for the detection of feline leukemia virus (FeLV) in 90 clinically ill cats from Bangkok, Thailand.

No	ICA	RT-qPCR		No	ICA	RT-qPCR		No	ICA	RT-qPCR	
	Result	Result	Ct		Result	Result	Ct		Result	Result	Ct
1	positive	positive	15.58	41	positive	positive	22.01	81	positive	positive	20.84
2	negative	positive	31.52	42	positive	positive	20.48	82	positive	positive	13.86
3	positive	positive	23.22	43	positive	positive	21.99	83	negative	positive	36.89
4	negative	negative	-	44	positive	negative	-	84	negative	negative	-
5	negative	positive	27.72	45	negative	negative	-	85	negative	positive	36.13
6	negative	positive	36.17	46	negative	negative	-	86	positive	positive	13.87
7	positive	positive	20.63	47	negative	positive	35.20	87	positive	positive	20.33
8	positive	positive	16.60	48	negative	negative	-	88	positive	positive	25.61
9	positive	positive	15.27	49	negative	negative	-	89	positive	positive	16.74
10	positive	positive	18.31	50	negative	negative	-	90	positive	positive	18.81
11	positive	positive	17.38	51	negative	negative	-				
12	positive	positive	25.49	52	negative	negative	-				
13	positive	positive	16.90	53	negative	negative	-				
14	positive	positive	17.51	54	negative	negative	-				
15	positive	positive	10.58	55	positive	positive	18.33				
16	positive	positive	14.68	56	negative	negative	-				
17	positive	positive	14.72	57	positive	positive	17.28				
18	positive	positive	17.07	58	positive	positive	11.86				
19	positive	positive	22.60	59	positive	positive	20.55				
20	positive	positive	18.27	60	positive	positive	18.58				
21	positive	positive	19.65	61	positive	positive	13.73				
22	positive	positive	21.64	62	negative	positive	32.33				
23	positive	positive	15.93	63	negative	negative	-				
24	positive	positive	32.08	64	negative	negative	-				
25	positive	positive	19.06	65	negative	negative	-				
26	positive	positive	18.92	66	negative	negative	-				
27	positive	positive	18.27	67	negative	negative	-				
28	positive	positive	19.06	68	negative	negative	-				
29	positive	positive	18.09	69	negative	negative	-				
30	negative	negative	-	70	negative	negative	-				
31	positive	positive	21.05	71	negative	negative	-				
32	positive	positive	19.14	72	negative	negative	-				
33	positive	positive	15.49	73	positive	positive	17.58				
34	negative	negative	-	74	negative	negative	-				
35	negative	negative	-	75	negative	negative	-				
36	negative	negative	-	76	negative	positive	29.15				
37	positive	positive	22.70	77	positive	positive	18.81				
38	positive	positive	17.95	78	positive	positive	13.12				
39	positive	positive	20.86	79	positive	positive	20.74				
40	positive	positive	12.03	80	positive	positive	20.11				

Ct: cycle threshold; positive: detected antigen or nucleic acid; negative: undetected antigen or nucleic acid.

**Table 2:** Comparative raw data of immunochromatographic assay (ICA) and reverse transcription quantitative polymerase chain reaction (RT-qPCR) for the detection of feline immunodeficiency virus (FIV) in 90 clinically ill cats from Bangkok, Thailand.

No	ICA	RT-qPCR		No	ICA	RT-qPCR		No	ICA	RT-qPCR	
	Result	Result	Ct		Result	Result	Ct		Result	Result	Ct
1	positive	positive	34.05	41	negative	negative	-	81	negative	negative	-
2	positive	positive	31.15	42	negative	negative	-	82	negative	negative	-
3	positive	positive	26.72	43	negative	negative	-	83	negative	negative	-
4	positive	negative	-	44	negative	negative	-	84	negative	negative	-
5	positive	positive	29.05	45	negative	negative	-	85	negative	negative	-
6	positive	positive	35.47	46	negative	negative	-	86	positive	positive	33.73
7	negative	negative	-	47	negative	negative	-	87	positive	positive	23.28
8	negative	negative	-	48	negative	negative	-	88	negative	positive	26.20
9	negative	negative	-	49	negative	negative	-	89	negative	positive	28.28
10	negative	negative	-	50	positive	positive	31.67	90	positive	positive	30.22
11	negative	negative	-	51	positive	negative	-				
12	negative	positive	26.40	52	positive	negative	-				
13	negative	negative	-	53	positive	negative	-				
14	negative	negative	-	54	positive	positive	30.82				
15	negative	positive	34.43	55	positive	positive	29.97				
16	negative	negative	-	56	positive	positive	30.51				
17	negative	negative	-	57	negative	positive	30.35				
18	negative	negative	-	58	negative	negative	-				
19	negative	negative	-	59	negative	negative	-				
20	negative	negative	-	60	negative	negative	-				
21	negative	negative	-	61	negative	negative	-				
22	negative	negative	-	62	negative	negative	-				
23	negative	negative	-	63	negative	negative	-				
24	negative	negative	-	64	negative	negative	-				
25	negative	negative	-	65	positive	positive	30.89				
26	negative	negative	-	66	positive	negative	-				
27	negative	negative	-	67	positive	negative	-				
28	negative	negative	-	68	positive	negative	-				
29	negative	negative	-	69	positive	positive	35.61				
30	negative	negative	-	70	positive	positive	36.42				
31	negative	negative	-	71	positive	positive	30.32				
32	negative	negative	-	72	positive	positive	34.85				
33	negative	negative	-	73	positive	positive	29.50				
34	negative	negative	-	74	positive	positive	30.43				
35	negative	negative	-	75	positive	positive	33.20				
36	negative	negative	-	76	positive	positive	25.89				
37	negative	negative	-	77	negative	positive	29.66				
38	negative	positive	31.39	78	negative	negative	-				
39	positive	positive	34.80	79	negative	negative	-				
40	negative	negative	-	80	negative	negative	-				

Ct: cycle threshold; positive: detected antibody or nucleic acid; negative: undetected antibody or nucleic acid.

**Table 3:** Comparative raw data of RNase hybridization-assisted amplification (RHAM) assay and reverse transcription quantitative polymerase chain reaction (RT-qPCR) for the detection of feline leukemia virus (FeLV) in 90 clinically ill cats from Bangkok, Thailand.

No	RHAM	RT-qPCR		No	RHAM	RT-qPCR		No	RHAM	RT-qPCR	
	Result	Result	Ct		Result	Result	Ct		Result	Result	Ct
1	positive	positive	15.58	41	positive	positive	22.01	81	positive	positive	20.84
2	positive	positive	31.52	42	positive	positive	20.48	82	positive	positive	13.86
3	positive	positive	23.22	43	positive	positive	21.99	83	positive	positive	36.89
4	negative	negative	-	44	negative	negative	-	84	positive	negative	-
5	positive	positive	27.72	45	negative	negative	-	85	negative	positive	36.13
6	negative	positive	36.17	46	negative	negative	-	86	positive	positive	13.87
7	positive	positive	20.63	47	negative	positive	35.20	87	positive	positive	20.33
8	positive	positive	16.60	48	negative	negative	-	88	positive	positive	25.61
9	positive	positive	15.27	49	negative	negative	-	89	positive	positive	16.74
10	positive	positive	18.31	50	negative	negative	-	90	positive	positive	18.81
11	positive	positive	17.38	51	negative	negative	-				
12	positive	positive	25.49	52	negative	negative	-				
13	positive	positive	16.90	53	negative	negative	-				
14	positive	positive	17.51	54	negative	negative	-				
15	positive	positive	10.58	55	positive	positive	18.33				
16	positive	positive	14.68	56	negative	negative	-				
17	positive	positive	14.72	57	positive	positive	17.28				
18	positive	positive	17.07	58	positive	positive	11.86				
19	positive	positive	22.60	59	positive	positive	20.55				
20	positive	positive	18.27	60	positive	positive	18.58				
21	positive	positive	19.65	61	positive	positive	13.73				
22	positive	positive	21.64	62	positive	positive	32.33				
23	positive	positive	15.93	63	positive	negative	-				
24	negative	positive	32.08	64	negative	negative	-				
25	positive	positive	19.06	65	negative	negative	-				
26	positive	positive	18.92	66	negative	negative	-				
27	positive	positive	18.27	67	negative	negative	-				
28	positive	positive	19.06	68	negative	negative	-				
29	positive	positive	18.09	69	negative	negative	-				
30	negative	negative	-	70	negative	negative	-				
31	positive	positive	21.05	71	negative	negative	-				
32	positive	positive	19.14	72	negative	negative	-				
33	positive	positive	15.49	73	positive	positive	17.58				
34	negative	negative	-	74	negative	negative	-				
35	negative	negative	-	75	negative	negative	-				
36	negative	negative	-	76	positive	positive	29.15				
37	positive	positive	22.70	77	positive	positive	18.81				
38	positive	positive	17.95	78	positive	positive	13.12				
39	positive	positive	20.86	79	positive	positive	20.74				
40	positive	positive	12.03	80	positive	positive	20.11				

Ct: cycle threshold; positive: detected nucleic acid; negative: undetected nucleic acid.

**Table 4:** Comparative raw data of RNase hybridization-assisted amplification (RHAM) assay and reverse transcription quantitative polymerase chain reaction (RT-qPCR) for the detection of feline immunodeficiency virus (FIV) in 90 clinically ill cats from Bangkok, Thailand.

No	RHAM	RT-qPCR		No	RHAM	RT-qPCR		No	RHAM	RT-qPCR	
	Result	Result	Ct		Result	Result	Ct		Result	Result	Ct
1	positive	positive	34.05	41	negative	negative	-	81	negative	negative	-
2	negative	positive	31.15	42	negative	negative	-	82	negative	negative	-
3	positive	positive	26.72	43	negative	negative	-	83	negative	negative	-
4	negative	negative	-	44	negative	negative	-	84	negative	negative	-
5	positive	positive	29.05	45	negative	negative	-	85	negative	negative	-
6	negative	positive	35.47	46	negative	negative	-	86	positive	positive	33.73
7	negative	negative	-	47	negative	negative	-	87	positive	positive	23.28
8	negative	negative	-	48	negative	negative	-	88	positive	positive	26.20
9	negative	negative	-	49	negative	negative	-	89	positive	positive	28.28
10	negative	negative	-	50	positive	positive	31.67	90	positive	positive	30.22
11	negative	negative	-	51	negative	negative	-				
12	positive	positive	26.40	52	negative	negative	-				
13	negative	negative	-	53	negative	negative	-				
14	negative	negative	-	54	positive	positive	30.82				
15	negative	positive	34.43	55	positive	positive	29.97				
16	negative	negative	-	56	positive	positive	30.51				
17	negative	negative	-	57	positive	positive	30.35				
18	negative	negative	-	58	negative	negative	-				
19	negative	negative	-	59	negative	negative	-				
20	negative	negative	-	60	negative	negative	-				
21	negative	negative	-	61	negative	negative	-				
22	negative	negative	-	62	negative	negative	-				
23	negative	negative	-	63	negative	negative	-				
24	negative	negative	-	64	negative	negative	-				
25	negative	negative	-	65	positive	positive	30.89				
26	negative	negative	-	66	negative	negative	-				
27	negative	negative	-	67	negative	negative	-				
28	negative	negative	-	68	negative	negative	-				
29	negative	negative	-	69	negative	positive	35.61				
30	negative	negative	-	70	negative	positive	36.42				
31	negative	negative	-	71	positive	positive	30.32				
32	negative	negative	-	72	negative	positive	34.85				
33	negative	negative	-	73	positive	positive	29.50				
34	negative	negative	-	74	negative	positive	30.43				
35	negative	negative	-	75	positive	positive	33.20				
36	negative	negative	-	76	positive	positive	25.89				
37	negative	negative	-	77	positive	positive	29.66				
38	positive	positive	31.39	78	negative	negative	-				
39	positive	positive	34.80	79	negative	negative	-				
40	negative	negative	-	80	negative	negative	-				

Ct: cycle threshold; positive: detected nucleic acid; negative: undetected nucleic acid.

**Table 5:** Summary of results comparing the performance of the immunochromatographic assay (ICA) and RNase hybridization-assisted (RHAM) test kit for the detection of Feline immunodeficiency virus (FIV) and Feline leukemia virus (FeLV), using reverse transcription quantitative polymerase chain reaction (RT-qPCR) as the reference standard.

		RT-qPCR			
		FeLV		FIV	
		Positive	Negative	Positive	Negative
ICA	Positive	53	1	22	7
	Negative	8	28	7	54
RHAM	Positive	57	1	22	0
	Negative	4	28	7	61

**Table 6:** Analytical sensitivity, specificity, accuracy, precision, and corresponding 95% confidence intervals (CIs) of the immunochromatographic assay (ICA) and RNase hybridization-assisted (RHAM) test kit for the detection of Feline immunodeficiency virus (FIV) and Feline leukemia virus (FeLV), using reverse transcription quantitative polymerase chain reaction (RT-qPCR) as the reference standard.

	ICA Compared RT-qPCR		RHAM Compared RT-qPCR	
	FeLV	FIV	FeLV	FIV
Sensitivity (95% Cl)	86.89% (82.24–90.91)	75.86% (66.25–84.15)	93.44% (90.12–96.11)	75.86% (66.25–84.15)
Specificity (95% Cl)	96.55% (94.34–98.04)	88.52% (82.32–93.72)	98.28% (96.22–99.35)	100.00% (100.00–100.00)
Accuracy (95% Cl)	90.00% (85.96–92.82)	84.44% (79.24–89.44)	94.44% (91.22–96.32)	92.22% (88.61–94.72)
Precision (95% Cl)	98.15% (95.67–99.19)	75.86% (66.25–84.15)	98.28% (96.22–99.35)	100.00% (100.00–100.00)

## Data Availability

The data presented in this study are available free of charge for any user on request from the corresponding authors.
